# Underlying motivations hampering Flemish primary care physicians from overcoming the barriers in osteoporosis care: an EMR-facilitated clinical reasoning study

**DOI:** 10.1186/s12913-023-10441-7

**Published:** 2023-12-16

**Authors:** Caroline Verdonck, Ellis Van Daele, Ruben Willems, Liesbeth Borgermans, Pauline Boeckxstaens

**Affiliations:** Department of Public Health and Primary Care, Faculty of Medicine and Health Sciences, Ghent University, Corneel Heymanslaan 10 – Entrance 42 – 4thFloor, 9000 Ghent, Belgium

**Keywords:** Multimorbidity, Primary care, Primary care physicians, Health priorities, Clinical decision, Making, Qualitative research, Osteoporosis

## Abstract

**Background:**

Over half of the European population aged minimum 65 years presents with at least two chronic diseases. Attention towards these diseases exhibits disparities, with limited primary care physician (PCP) attention for osteoporosis. This was confirmed in a previous integrated osteoporosis care (IOC) project in which notable difficulties to enlist PCPs arose. Consequently, this study was initiated in Flemish PCPs for in-depth analysis of underlying mechanisms hampering PCPs to fully commit to osteoporosis care.

**Methods:**

A qualitative Electronic Medical Record (EMR)-facilitated clinical reasoning study was conducted. A semi-structured interview guide was employed to guide PCPs from reflections on their own patients to broader views regarding osteoporosis care. An inductive thematic analysis was performed using NVivo 12.

**Results:**

Thirteen PCPs were interviewed. They stated that osteoporosis patients often had complex (medical) profiles. PCPs emphasised the ongoing necessity for prioritisation within this context. This leads to a competition for PCP attention during consultations at three levels: i. between acute versus preventive care; ii. between primary fracture prevention and other preventive services and iii. between secondary fracture prevention and other preventive services; spanning eight areas of competition: disease significance, perceived impact, PCP awareness, the patient agenda, PCP competence, PCP support, perceived patient burden, and efficiency of care provision. Applicability of these areas of competition differed between levels.

**Conclusion:**

The intricate context in which PCPs operate, creates a competition for PCP attention leading to a lack of attention for fracture prevention. To preserve efforts in fracture prevention, areas of competition should be systematically addressed.

**Trial registration:**

Approval for the study has been provided by the Ghent University Hospital Ethics Committee (BC-09797).

**Supplementary Information:**

The online version contains supplementary material available at 10.1186/s12913-023-10441-7.

## Background

Over half of the European population aged 65 year or older presents with a minimum of two chronic diseases, and this proportion is expected to rise [1]. The allocation of attention among diverse chronic diseases exhibits marked disparities, with a predominant focus on diabetes mellitus (DM), ischemic heart disease, stroke, and chronic obstructive pulmonary disease (COPD) [2], whilst the level of awareness regarding conditions such as osteoporosis remains limited [3]. Osteoporosis is a chronic metabolic condition impacting bone mineral density (BMD) and predisposing individuals to fragility fractures [4]. In line with the management of most chronic conditions, primary care (PC) is considered the cornerstone for identifying and managing high-risk patients. However, osteoporosis and fracture prevention tend to be overlooked in the clinical practice of primary care physicians (PCPs). Studies have indicated that only 0.9% to 29.0% of eligible patients – considering Belgian reimbursement criteria^1^ (Additional file 1) – undergo BMD testing [5]. Moreover, less than 25% high-risk patients receive pharmacological treatment [6]. Despite the perpetual evolution and occasional contradictions within clinical guidelines, in contrast to the 40% to 80% coverage of high-blood pressure patients with anti-hypertensive medication [7], osteoporosis commands relatively little attention in PC practice.

In an effort to enhance the quality of osteoporosis care for postmenopausal women at high risk of fractures, an integrated osteoporosis care (IOC) initiative was established in Ghent, Belgium [8]. The project was designed to address the well-documented barriers in osteoporosis care [9–12]. Its strategic approach encompassed two key components: i. the introduction of dedicated osteoporosis nurses tasked with patient education, empowerment and follow-up and ii. comprehensive, one-on-one educational sessions for PCPs. These sessions covered various aspects, including case-finding, diagnostic and supplementary assessments, (non-)pharmacological treatment options, treatment objectives, medication side effects and adverse events, and follow-up, as well as referral criteria for paramedical and specialist care, spanning both primary and secondary fracture prevention. The former pertains to the prevention of an index fracture, while the latter concerns refracture prevention.

During the recruitment phase of the IOC project, notable difficulties arose in enlisting PCPs willing to engage in the research project. Hence, notwithstanding efforts to counterbalance the well-known barriers in osteoporosis care, unknown factors hampered PCPs engagement.

Consequently, qualitative research was initiated in Flemish PCPs for in-depth analysis of underlying mechanisms hampering PCPs to fully commit to osteoporosis care.

## Methods

### Study design

Semi-structured interviews were conducted between June and September 2021. The interview guide (Additional file 2) was crafted to guide PCPs from reflections on their own clinical practice to broader views regarding osteoporosis care. Question development was informed by recognized disparities in attention for chronic diseases [6, 7].

In-depth interviews were conducted in Dutch by CV at the respective PCPs’ offices, employing an Electronic Medical Record (EMR)-facilitated clinical reasoning approach. PCPs were tasked to searching their EMR database for patients who had received an osteoporosis diagnosis, experienced a fragility fracture, and/or were undergoing pharmacological anti-osteoporosis treatment. To mitigate the potential for recall bias, discussions centred on the two most recently attended eligible patients, with no limitations on when these consultations had occurred. Subsequently, broader views on osteoporosis care were explored. Throughout the interviews, participant checking was carried out. All interviews were recorded and transcribed at verbatim, and quotations extracted for the reporting of the results forward–backward translated to English by CV and RW. Data-collection continued until data saturation was achieved, with interim analyses conducted every two or three interviews. An inductive thematic analysis was applied to each practice type using a step-wise approach, led by two senior (PB and RW) alongside two junior researchers (CV and EVD). During an initial analysis meeting, themes identified during independent readings were discussed collectively. Thereafter, junior researchers independently analysed the subsequent interviews using NVivo 12 for Windows. Upon the completion of all interviews, the results were once more discussed by all researchers to initiate the development of a thematic tree through a comprehensive approach. This iterative process, incorporating both holistic and detailed analysis is referred to as hermeneutic circle leading to a hermeneutic spiral [13], and was repeated thrice until a final thematic tree could be drawn. This thematic tree was subjected to content analysis to formulate a descriptive narrative elucidating the mechanisms hampering PCPs’ full commitment to osteoporosis care.

### Participants

The research was performed in PCPs previously invited to participate in an IOC in PC project [8], there were no exclusion criteria. PCPs were categorized based on i. intervention (IG) or control group (CG) and ii. activity level: high activity – including at least three patients – and low activity – including maximum two patients. Non-participants, who refused participation in the intervention study, were also invited.

Approval for the study has been provided by the Ghent University Hospital Ethics Committee (BC-09797). All methods were carried out in accordance with the Declaration of Helsinki. All participants voluntarily signed an informed consent form after receiving both written and oral information on the study and prior to participation.

## Results

Data saturation was attained following 13 PCP interviews. A summary of PCP characteristics and interview duration can be found in Table 1.

**Table 1:** PCP characteristics and interview duration

	**Gender**	**Age (y)**	**Practice form**	**PCP group**	**Interview duration**
PCP1	Male	60	Multidisciplinary group practice	CG LA	52′42"
PCP2	Female	30	Group practice	IG LA	37′03"
PCP3	Male	60	Multidisciplinary group practice	CG LA	1h05′05"
PCP4	Male	41	Group practice	CG HA	50′18"
PCP5	Female	53	Solopractice	CG LA	46′10"
PCP6	Female	34	Community health centre	NP	49′12"
PCP7	Female	50	Community health centre	NP	47′53"
PCP8	Female	33	Group practice	CG HA	59′24"
PCP9	Male	57	Solopractice	IG HA	1h03′26"
PCP10	Male	84	Group practice	IG HA	1h01′33"
PCP11	Female	31	Multidisciplinary group practice	NP	52′45"
PCP12	Female	31	Multidisciplinary group practice	IG HA	41′00"
PCP13	Female	35	Multidisciplinary group practice	IG HA	40′05"
**Average**		**46.1**			**51′17"**

*CG* Control group in the IOC project, *HA* Recruitment of 3 or more patients in the IOC project, *IG* Intervention group in the IOC project, *LA* Recruitment of 0 to 2 patients in the IOC project, *NP* Non-participant in the IOC project

A predominant theme consistently identified throughout the interviews was the presence of competition for PCP attention during consultations, which appears to constitute the primary mechanism hampering PCPs commitment to osteoporosis care. Patients eligible for reflection were often patients with complex (medical) profiles. PCPs emphasised the ongoing necessity for prioritisation within the context of multimorbidity, requiring constant weighing of sometimes conflicting interests. Three levels of competition for PCP attention were discerned: the tension between i. acute versus preventive care, ii. primary fracture prevention versus other preventive services and iii. secondary fracture prevention versus other preventive services. These competitive dynamics translated to limited case identification and deviations from guideline recommendations for patients at high risk of fragility fracture. It is worth noting that views on and management of osteoporosis did not differ based on type of practice. In the ensuing paragraphs these levels of competition are explored in depth.

### Acute versus preventive care

**Fig. 1:**
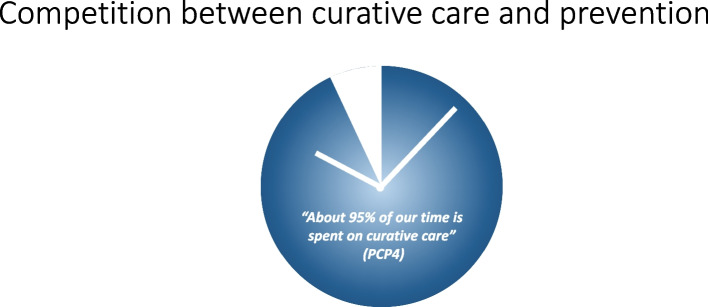
Competition between curative and preventive services in primary care

PCPs acknowledge the importance of prevention and their role herein. However, they state that consultations typically revolve around specific patient inquiries or complaints such as acute issues or follow-up of chronic diseases. Attending to these needs constitutes PCPs’ primary objective: *“Why does this patient consult me now? That question needs to be answered.”(PCP1)*. Consequently, time for preventive measures becomes constrained (Fig. 1). This patient-driven agenda represents the sole area of competition identified at this level.

### Primary fracture prevention

**Fig. 2:**
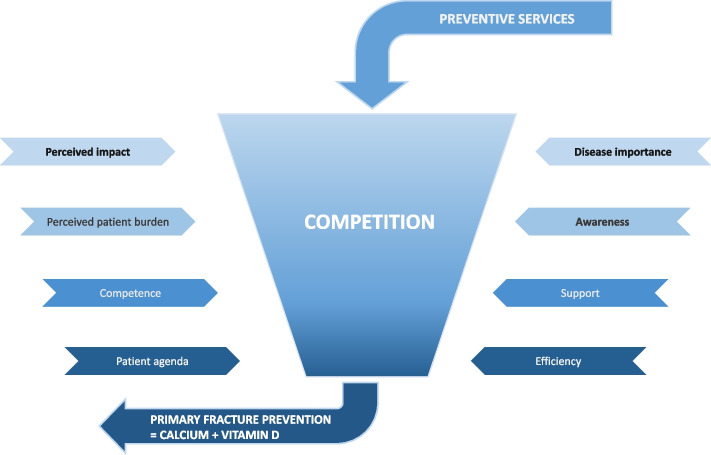
Areas of competition in primary and secondary preventive services affecting fracture prevention. All areas of competition identified apply to primary fracture prevention. In secondary fracture prevention, only those areas of competition marked in bold are applicable.

Although PCPs expressed feeling responsible for delivering primary fracture prevention, it rarely finds its way onto the consultation agenda due to the presence of competing priorities with other preventive services. At the level of primary fracture prevention, eight distinct areas of competition were identified (Fig. 2): disease significance, PCPs’ perceived impact, the patient’s agenda, PCP awareness, PCP competence, PCP support, perceived burden of care, and efficiency. Although not all areas of competition were pertinent to every physician, no divergent perspectives were observed within the respective areas.

#### Disease significance

PCPs acknowledge the significance of fracture prevention: *“If she falls and fractures. That’s not beneficial to her health…” (PCP12)*. However, they accord higher priority to the prevention of other diseases: *“I think it is important. But other things are more important.” (PCP4)*. Osteoporosis is not perceived a real disease: *“Osteoporosis… whether you can regard it as a disease? A tricky question.”(PCP10)*. Moreover, its significance is further diminished by the absence of public health campaigns and its omission from the Domus Medica^2^ (DM) health guide^3^:*”Our opinion leaders do not really perceive it as a relevant issue.”(PCP3)*.

#### Perceived impact

The significance of PCPs’ work is rooted in the potential impact on patients’ well-being, and other diseases and medical procedures are perceived to generate more substantial impact: *“What I find interesting in preventive medicine? Preventive testing, blood samples, cancer prevention. This has a very high impact if you detect these in an early stage.”(PCP4).* Doubt surrounding the effectiveness of treatment further diminish the perceived impact: *“The drugs are not 100% effective and consequently, the outcome, is rather limited.”(PCP10)*. This scepticism is compounded by a lack of a sense of urgency: *“Osteoporosis is of course never an acute danger.”(PCP3)*. However, the perceived impact of primary fracture prevention is subject to patient age: *“Maybe I am more inclined to do this [dual-energy x-ray absorptiometry (DXA) referral] in younger women than at age 77.”(PCP13)*.

#### Patient agenda

PCPs elaborated on the difficulties of associated with broaching fracture prevention: *“It is about what the patient brings to the table: ‘This is what reduces my quality of life most.’”(PCP1)*. They noted patients’ unawareness contributing to the low priority accorded to osteoporosis in the patient agenda: *“Patients consult for their knee, a common cold, a blood sample analysis. They don’t consult with ‘doctor, could I have osteoporosis?’.”(PCP3)*. PCPs attributed this lack of awareness to the asymptomatic nature of osteoporosis: *“The tragedy of osteoporosis is that it is a silent disease.”(PCP1)*. However, the acknowledged that fractures don’t necessarily increase awareness: *“I don’t think they link them. That if someone broke a hip that it could possibly be due to osteoporosis.”(PCP5)*.

Additionally, patients’ perspectives on treatment play a role: *“Some people are reluctant to the costs of medication. Then I question the usefulness of performing a DXA.”(PCP6).* Furthermore, there is only limited societal awareness of osteoporosis: *“Osteoporosis isn’t sexy. It’s not present in the media.”(PCP9).*

#### PCP awareness

Primary fracture prevention doesn’t occupy a prominent position in the PCPs’ minds: *“Probably it is not front and center in my head with all the things you do or think about in prevention.” (PCP12)*. Unlike for many other diseases, there are close to no established habits for early detection of high-risk patients for osteoporosis: *“To ask questions that might indicate osteoporosis, you don’t do that automatically”(PCP9).* In contrast to other diseases often presenting with ‘entry points’, such as symptoms, that facilitate the incorporation of preventive measures, the silent nature of osteoporosis limits PCP awareness: *“Those entry points just make life way easier.”(PCP6)*.

#### Competence

Relative to prevention strategies for other diseases, PCPs express a lesser degree of confidence in their competence regarding primary fracture prevention: *“Probably my knowledge for other diseases’ management is better.”(PCP12)*. Osteoporosis is only touched upon limitedly during education and internships: *“This is, at least partially, due to the fact that it isn’t really provided in our basic education. You know something but not really… mastering it.”(PCP6)*. This is aggravated by the perceived lack of accessible and visible information thereafter: *“What holds me back herein? I think the accessibility of information, and visibility.”(PCP6)*.

#### Support

While *“thinking about it.”(PCP8)* remains the primary approach for osteoporosis, comprehensive ‘support systems’ are in place for attending to other comorbidities. PCPs encounter a deficiency of clear guidelines enabling proactive identification of high-risk patients: *“I miss something that allows me to identify patients prior to their first fracture”(PCP5)*. This contrasts markedly with the care pathways that have been developed for other diseases: *“For diabetes we have these care pathways that support us.”(PCP2)*. Furthermore, the EMR offers reminders for monitoring various diseases, but lacks prompts for fracture prevention: *“In the EMR, we are reminded: ‘you need to do this, you need to do that’, but osteoporosis is not included.”(PCP11)*. In addition, there is a dearth of communication tools available to explain osteoporosis to patients: *“The advantage of cardiovascular prevention or breast cancer screening is that you have tools to explain this to patients. But not for fracture prevention.”(PCP6)*. Moreover, osteoporosis screening is not included in public health campaigns, despite availability of such campaigns for other diseases: *“Women are being called upon for breast cancer screening. Why can’t they receive an invitation for BMD testing?”(PCP4)*.

#### Perceived burden of care

PCPs reported heightened awareness of the substantial burden of care borne by patients with complex health profiles. The presence of polypharmacy in these patients deters PCPs from introducing additional medication: *“That’s again an additional item to the sometimes long lists of medication. Knowing that alendronate provides side effects quite frequently… “(PCP13)*. The issue is compounded by the fact that calcium and vitamin D are not reimbursed:*”Calcium and vitamin D are not cheap… And you can’t prescribe just alendronate.”(PCP13)*. Additionally, PCPs perceived the request for DXA imposing both a practical and financial burden: *“We do one blood sample analysis here and we have all the results. That costs €8. To ask a patient to pay €55 for only osteoporosis screening is a bridge too far.”(PCP9).* This sentiment is accentuated by doubts concerning the utility of osteoporosis screening: *“The usefulness of screening, that’s relative.”(PCP4)*.

#### Efficiency of care provision

In contrast to the organisation of screening for other comorbidities, organising osteoporosis screening poses considerable challenges, as it cannot be combined with other screening services: *“In a preventive consultation we do a lot in very limited time. Vaccination, blood samples, medication review… And DXA falls by the wayside because of the separate request form.”(PCP4)*. PCPs stated that *“If there was a blood test for BMD everyone would do it.”(PCP12)*.

The aforementioned areas of competition have led PCPs to deviate from the principles of evidence-based medicine (EBM) to address primary fracture prevention: *“In a blood sample analysis we also evaluate vitamin D. When low I anticipate osteoporosis and ‘treat’ them blindly with calcium and vitamin D.”(PCP9)*, and to provide lifestyle advice spanning various domains of health and well-being.

### Secondary fracture prevention

Engagement in secondary fracture prevention encounters challenges arising from competing priorities associated with other comorbidities as well. However, only three areas of competition have been identified: disease significance, PCP awareness and perceived impact (Fig. 2). The latter exerts most substantial impact on PCP decision-making the most, and is particularly sensitive to the patient’s profile.

#### Disease significance

Even in patients who have experienced a fracture, osteoporosis is perceived a relatively benign disease in comparison to other comorbidities: *“Preferably you don’t fracture your hip, but most people with a hip prothesis are really well off, whilst a diabetic foot… you can’t add a new toe”(PCP13)*.

#### PCP awareness

Fractures are infrequently associated with osteoporosis: *“There is this fracture and then you need to consider ‘why?’. Not solely because of the fall, but because she might have osteoporosis. And that’s apparently a step too far.”(PCP12)*. It is suggested that this responsibility should primarily rest with Emergency Department personnel, as patients most frequently present with fractures there: *“The orthopaedic surgeons see those low-impact fractures arriving, they should say ‘when this is healed we perform a DXA’.”(PCP4)*.

#### Perceived impact

Similar to primary fracture prevention, PCPs seek to make a meaningful impact. As fractures are often viewed as benign, the use of medication to prevent them is regarded as redundant: *“If you look at osteoporosis treatment recommendations, guidelines state ‘Provide lifestyle advice’… That’s what we do.” (PCP7)*. Moreover, anti-osteoporosis medication is perceived as having limited effectiveness: *“A hip fracture is a serious problem, but I question the extent to which we can prevent that with treatment.”(PCP13),* and PCPs cited a lack of access to up-to-date evidence on pharmacological treatment: *“I still look at my powerpoint of 4 years ago. The information is not really up-to-date.”(PCP6)*. Furthermore, PCPs often felt helpless when faced with patients dealing with complex socio-economic situations and/or multiple comorbidities: *“When I see her, my heart sinks into my boots. What can I do for such a patient?”(PCP1)*. Secondary fracture prevention was perceived as more feasible for individuals with fewer comorbidities or a more stable health status: *“Aside from his COPD, which was under control, there was little. So the osteoporosis, we could do something with.”(PCP6)*.

## Discussion

Our study scrutinized that (absence of) attention to fracture prevention is primarily driven by the intricate multimorbid context in which PCPs operate. This complex environment engenders a competition for attention during consultations that fundamentally shapes the contours of the consultation agenda. This may elucidate why, notwithstanding our intentions, engaging PCPs in the IOC project proved to be challenging. Our interviews provided evidence for this competition manifesting at three distinct levels: i. acute versus preventive care, ii. primary fracture prevention versus the prevention of other medical conditions and iii. secondary fracture prevention versus preventive services for other diseases. Eight areas of competition were identified, all of which are pertinent to the second level of competition, while three persist within the third. There was unequivocal consensus among PCPs across all levels and areas of competition, underscoring the deeply ingrained clinical inertia in osteoporosis care.

The first level of competition arises from tension between PCPs’ primary objective to address the immediate concerns presented by patients and allocating time to prevention. In our study, PCPs contented little patient interest for prevention, a viewpoint consistent with findings from Mirand et al. [14], although diverging from our own research involving patients [15]. Furthermore, our findings underscore the challenge faced by PCPs to execute their mandate: in the current context, there is insufficient time to address all preventive services while simultaneously attending to acute care needs and overseeing the monitoring and follow-up of chronic diseases.

Complying with all preventive recommendations would extend a PCP’s working day with 7.4 h [16, 17]. Consequently, competition for preventive services amid various medical conditions is insuperable. Eight areas of competition were identified, with all being relevant in primary prevention and three applying in secondary prevention. While certain areas of this competition have been previously documented, their relation to the multimorbid context in which osteoporosis management is situated has not been previously explored.

The perception of osteoporosis not being a real disease [9, 10], PCP’s lack of confidence in their own competence [9, 10, 18–20], absence of a sense of urgency [9, 19], and limited perceived impact [10, 19] have been previously documented. In our study, the latter was subject to patient characteristics, particularly age, as younger patients were more inclined to receive primary fracture prevention. Furthermore, PCPs regarded osteoporosis management, encompassing both the procurement of DXA and pharmacological treatment, as unduly burdensome for patients, corroborating earlier research [10, 19]. Newly identified challenges at this level of competition impeding the uptake of fracture prevention include limited PCP support and care provision inefficiency. The delivery of both primary and secondary fracture prevention predominantly relies on PCP attention, which is known [10, 19] and confirmed to be low. In PCPs’ opinion, the absence of symptoms (prior to a fracture) results in osteoporosis being absent in the patient agenda. This is compounded by public unawareness leading to the absence of health campaigns, support tools, availability of communication tools, etc. Moreover, addressing primary fracture prevention is perceived inefficient. While for many other conditions case-finding can be combined using one blood sample analysis, the diagnostic gold standard for osteoporosis remains DXA [3], requiring additional PCP effort.

Nonetheless, PCPs assumed responsibility for addressing primary fracture prevention, leading them to adopt a strategy of ‘choosing the path of least resistance’, which involved commencing treatment with calcium and vitamin D without thorough evaluation.

After an index fracture, the number of areas of competition diminishes, yet the perception of osteoporosis is equally depreciatory: other co-morbidities are appraised as more urgent, and/or having more significant repercussions. These observations have been previously described as factors orienting prioritisation of health services in complex patients [21]. In comparison to primary fracture prevention, the patient profile assumes a more dominant role in clinical decision-making, with PCPs primarily adhering to the principle of ‘primum non nocere’ in older or more complex patients. Consequently, they often forgo secondary fracture prevention, aligning with recent research [21].

Our findings suggest that various areas of competition reinforce one another, as for some routinely applied preventive measures the provided rationale is equally true. For instance, a perceived lack of urgency could be applicable to hypertension too. However, literature indicates a notably higher emphasis on pharmacological primary cardiovascular prevention compared to pharmacologically preventing index fractures [6, 7]. This difference in attention might be attributed to several factors, including hypertension’s presence in the patient agenda, its integration in PCP routines, the ease of identification, and so forth. Similarly, the silent nature of osteoporosis is shared with numerous primary prevention areas, such as hypercholesterolaemia. Nevertheless, cholesterol level assessments are frequently requested by patients. Lastly, neither patients nor PCPs consistently associate fractures with osteoporosis, even though fractures are an overt expression of bone fragility, akin to how a myocardial infarction is consequential to atherosclerosis. These observations underscore awareness disparities, which partially elucidate why clinical inertia in osteoporosis care persists despite growing emphasis on chronic disease prevention.

Hence, in order to elevate fracture prevention to a level commensurate with preventive services for other conditions, addressing these areas of competition is imperative. This entails i. allocating sufficient resources and time to preventive services in general, aiming to diminish inter-disease competition for preventive services. Sharing and delegating these services between PCPs and other healthcare providers has demonstrated positive in fragility fracture prevention [22]. Furthermore, exploring the provision of integrated, non-disease-specific preventive services is warranted; ii. increasing the likelihood of fracture prevention featuring on the patient agenda by enhancing awareness for osteoporosis. Mass media campaigns have been proposed as a modifying strategy to mitigate the risk of non-communicable diseases [23] and have proven effective in reducing risk behaviour for cardiovascular disease [24]. However, research on the factors driving the patient’s consultation agenda may be needed, as solely addressing awareness may not suffice; iii. impeding as many barriers as possible to fracture prevention, such as offering DXA and osteoporosis treatment at an absolute minimum of out-of-pocket costs [15, 25]. Furthermore, it is worth exploring the utility of bone turnover markers as a diagnostic tool for osteoporosis. The cost-effectiveness of population screening has been demonstrated in the United States [26], and several countries have implementation population health campaigns [27]. Further research should investigate the cost-effectiveness of population screening within the context of the Belgian healthcare system; and iv. enhancing PCP competence and awareness, including the provision of a scientifically validated osteoporosis management guideline. Guideline use can bolster confidence in and execution of management strategies [28]. Additionally, improving PCP education and training in osteoporosis management and fracture prevention, through initiatives such as workshops facilitating the acquisition of necessary knowledge and competence in a condensed timeframe, should be prioritized [29].

Whilst our research offer valuable insights, it is important to acknowledge its limitations. First, osteoporosis treatment and fracture prevention were used intertwined, which may have influenced PCPs’ comprehension and responses. This was addressed by rigorously separating and analysing views on primary and secondary fracture prevention. Second, some participants were acquainted with the researcher from the previous IOC project, which may have introduced participant bias [30]. Therefore, an interview guide was employed and interviews were conducted in the PCP’s office, creating a more comfortable environment conductive to candid opinions. Third, the interviewer being a researcher in the field of osteoporosis could have influenced the interpretation of PCPs’ answers. This concerns has been mitigated by i. participant checking during the interviews, ii. a second researcher, primarily a PCP, independently analysing the interviews and iii. organizing analysis-meetings with senior researchers.

Despite these limitations, several notable strengths can be identified. To the best of our knowledge, this research represents the first comprehensive exploration of the underlying motivations in PCPs hampering their commitment to osteoporosis care. Additionally, this research recognizes osteoporosis as a co-morbid condition, a perspective grounded in the fact that 95% of osteoporosis patients have at least one co-existent medical condition [31]. Failing to consider this context would undermine our understanding of its impact on osteoporosis care. On top, in light of increasing prevalence of patients with complex health needs [1], the comprehension of competition between medical conditions may prove invaluable in shaping future healthcare reforms. Finally, by soliciting PCPs to evaluate their own patients, our study enabled them to engage in a more pronounced reflection on the contextual factors affecting the prioritisation process during consultations.

## Conclusion

The intricate context in which PCPs operate, creates a competition for PCP attention leading to a lack of attention for fracture prevention. This competition is manifest at three levels and spreads over eight areas of competition. To preserve efforts in (fracture) prevention, it is imperative to systematically address all these areas of competition.

### Supplementary Information

**Additional file 1.** Reimbursement criteria for DXA.

**Additional file 2.** Interview guide.

## Data Availability

The data used and analysed during the current study are available from osf.io/5z6eq (doi: 10.17605/OSF.IO/5Z6EQ).

## Abbreviations

BMD: Bone mineral density
COPD: Chronic obstructive pulmonary disease
DXA: Dual-energy X-ray absorptiometry
EMR: Electronic medical record
IOC: Integrated osteoporosis care
PC: Primary care
PCP: Primary care physician

## Footnotes

1 Eligibility has been based on reimbursement criteria as there is currently no scientifically validated guideline for the screening/case-finding and management of osteoporosis in Belgium.

2 Domus Medica is the largest PCP association in Flanders (Belgium).

3 The prevention guide is an evidence-guide to support PCPs in developing a tailored prevention strategy using questionnaires to calculate individualized risk profiles and provide tailored recommendations (https://www.domusmedica.be/expertisedomein/preventie-gezondheidspromotie/gezondheidsgids-domus-medica).
