# Supplementary material for: Underlying motivations hampering Flemish primary care physicians from overcoming the barriers in osteoporosis care: an EMR-facilitated clinical reasoning study
Source: BMC Health Serv Res. 2023 Dec 16;23:1428. doi: 10.1186/s12913-023-10441-7 (PMC10725585; doi:10.1186/s12913-023-10441-7)
Supplement: Supplementary file 1 — Additional file 1. Reimbursement criteria for DXA. [file 12913_2023_10441_MOESM1_ESM.docx]

Additional file 1: Reimbursement criteria for DXA

## DXA reimbursement based on related-related criteria

DXA is reimbursed for all women aged 65 years or older in the presence of a family history (1^st^ or 2^nd^ degree relatives) of hip fracture.

## DXA reimbursement based on non-age-related criteria

DXA is reimbursed regardless age in the presence of one or more of the following risk factors:

- a low-impact vertebral fracture of non-oncologic origin;
- antecedental peripheral low impact fracture(s), excluding fractures at the fingers, toes, skull, facial bones or cervical spine;
- prescribed corticosteroid therapy proloning over at least 3 consecutive months in a dosing regimen equivalent to or higher than 7.5mg prednisolone daily;
- oncological patients receiving anti-hormonal therapy or women with oncological-therapy induced menopause;
- patients with at least one of the following diseases:

1. rheumatoid arthritis;
2. non-stable and non-treated hyperthyroid disease;
3. hyperprolactinemia;
4. prolonged hypogonadism (including therapeutical orchidectomy or long-term treatment with gonadotrophine-releasing-hormones (GnHR);
5. renal hypercalciuria;
6. primary hyperparathyroid disease;
7. osteogenesis imperfecta;
8. Cushing’s disease/syndrome;
9. anorexia nervosa with BMI < 19kg/m²;
10. early menopause (<45y).

Reimbursement is foreseen every five years.
