# Supplementary material for: Underlying motivations hampering Flemish primary care physicians from overcoming the barriers in osteoporosis care: an EMR-facilitated clinical reasoning study
Source: BMC Health Serv Res. 2023 Dec 16;23:1428. doi: 10.1186/s12913-023-10441-7 (PMC10725585; doi:10.1186/s12913-023-10441-7)
Supplement: Supplementary file 2 — Additional file 2. Interview guide. [file 12913_2023_10441_MOESM2_ESM.docx]

Additional file 2: Interview guide

## Case discussion

I would like to start the interview with discussing some patient cases. Therefore, I would like to ask you to run the following search query^[[1]](#footnote-1)^ in your Electronic Medical Record (EMR): women, aged 50 years or older, meeting one of the following criteria:

- Patients with a registered diagnosis of osteoporosis OR
- Patients with registered fragility fractures OR
- Patients on anti-osteoporosis medication (bisphosphonates, denosumab, teriparatide or raloxifene).

*Once the search query has run, a list of patients in alphabetical order is provided to the physician, the first two patients will be discussed using the following questions as guidance.*

May I ask you to open the record of the first patient in your list?

1. Could you please provide me a short description of the patient, respecting her privacy (no names), including age, chronic comorbidities, important aspects of the medical history, chronic medication use, social context if deemed important?
2. Which steps have been taken with regards to fracture prevention?
3. Which aspects have been decisive factors in determining your actions with regards to whether or not investing in fracture prevention?
4. Are these aspects different from those that impact on your decision to invest in other chronic morbidities^[[2]](#footnote-2)^?
5. Why are these aspects different/similar?
6. Which choices regarding (not) initiating fracture prevention are decided in consultation with your patient and why?
7. Which choices regarding (not) initiating treatment for other chronic morbidities are decided in consultation with your patient and why?

## Postulations

I would like to discuss with you four postulations on fracture prevention and osteoporosis (care).

***Postulation 1: “Osteoporosis is a disease”***

- Which aspects are decisive in concluding osteoporosis is (not) a disease?
- What is your view in this matter concerning other chronic conditions such as heart failure, diabetes, hypertension, hypercholesterolemia, etc.?
- Where do differences or similarities in your view on these conditions on the one hand and osteoporosis on the other hand stem from?
- How do you think your patients view osteoporosis?
- How do you think your patients view osteoporosis in comparison to other chronic conditions?

***Postulation 2: “It is the GP’s responsibility to take up fracture prevention” (Connectedness)***

- Which are your responsibilities with regard to fracture prevention?
- Which are your patient’s responsibilities with regard to fracture prevention?
- (How) do you discuss these responsibilities with your patients?

***Postulation 3: “As a GP, I can prioritize in which themes enter the agenda during patient consultations” (autonomy)***

- Which aspects are decisive on your perception of (not) being able to prioritize the themes discussed during consultations?
- (How) affect these aspects your fracture prevention management?

***Postulation 4: “I feel competent to provide qualitative fracture prevention to my patients” (competence)***

- Which factors affect your competence perception?
- How does your competence perception for fracture prevention differ/is similar to your competence perception for managing other chronic conditions such as heart failure, diabetes, hypertension, hypercholesterolemia, etc.?
- Which aspects hold you back in increasing your competence in fracture prevention?
- To which extend and why is this different/similar for other chronic conditions?

## Reflection

Reflection on a) case studies and b) answers to the postulations:

The interviewer adopts a proactive attitude to discuss conflicting or unclear answers.

## Closing of the interview

I would like to thank you for your time, and your cooperation in this study. To end with, I would like to ask you two remaining questions.

1. May I ask you what your age is?
2. May I ask which kind of practice this is?

Thank you.

1. Search strategies for the most frequently used EMR software packages were provided to the family physicians upon request. [↑](#footnote-ref-1)
2. This question was only posed in case the patient suffered other comorbidities as well. [↑](#footnote-ref-2)
